# Immunomodulatory and antioxidant effects of Glycyrrhiza uralensis polysaccharide in Lohmann Brown chickens

**DOI:** 10.3389/fvets.2022.959449

**Published:** 2022-08-26

**Authors:** Hui Zhou, Chen Dai, Xuejie Cui, Tao Zhang, Yanyun Che, Kun Duan, Lei Yi, Audrey D. Nguyen, Nannan Li, Cristabelle De Souza, Xin Wan, Yu Wu, Kun Li, Yuhong Liu, Yi Wu

**Affiliations:** ^1^College of Veterinary Medicine, Institute of Traditional Chinese Veterinary Medicine, Nanjing Agricultural University, Nanjing, China; ^2^MOE Joint International Research Laboratory of Animal Health and Food Safety, College of Veterinary Medicine, Nanjing Agricultural University, Nanjing, China; ^3^Experimental Teaching Center of Life Science, College of Life Sciences, Nanjing Agricultural University, Nanjing, China; ^4^College of Pharmacy, Shandong University of Traditional Chinese Medicine, Jinan, China; ^5^Beijing Key Laboratory of Traditional Chinese Veterinary Medicine, Beijing University of Agriculture, Beijing, China; ^6^Engineering Laboratory for National Healthcare Theories and Products of Yunnan Province, College of Pharmaceutical Science, Yunnan University of Chinese Medicine, Kunming, China; ^7^China Tobacco Henan Industrial Co., Ltd, Zhengzhou, China; ^8^Department of Animal Science, Huaihua Polytechnic College, Huaihua, China; ^9^Department of Biochemistry and Molecular Medicine, Davis Medical Center, University of California, Sacramento, Sacramento, CA, United States; ^10^Department of Pathology, Stanford University, Palo Alto, CA, United States

**Keywords:** Glycyrrhiza uralensis polysaccharide, immunomodulatory effects, antioxidant activities, Newcastle disease, chickens

## Abstract

Glycyrrhiza polysaccharide extract 1 (GPS-1) is a bioactive component isolated from Glycyrrhiza uralensis, also known as Chinese licorice. It appears to be pharmacologically active as an antibacterial, antiviral, and anti-tumor agent. GPS-1 has also been shown to buffer liver health and regulate the immune system. Moreover, GPS-1 is low cost and easy to extract. More study was needed to elucidate the biochemical pathways underlying the immunomodulatory and antioxidant benefits observed in Glycyrrhiza polysaccharide extract 1 (GPS-1). *in vitro* experiments on chicken lymphocytes and dendritic cells (DCs) show that GPS-1 significantly promotes the proliferation of immune cells and is linked to lymphocytes' secretion of IL-12, IFN-γ, and TNF-α by. DC secretion of NO, IL-2, IL-1β, IFN-γ, TNF-α, and IL-12p70 was also increased significantly. Additionally, GPS-1 also displayed a significant antioxidant effect *in vitro*, able to scavenge DPPH, hydrogen peroxide, ABTS, and other free radicals like superoxide anions. Separately, GPS-1 was tested in vivo in combination with the Newcastle disease virus (NDV) – attenuated vaccine. 120 Lohmann Brown chickens were vaccinated, while another 30 became the unvaccinated blank control (BC) group. For three consecutive days 1 mL of GPS-1 was administered at doses of 19.53 μg/mL, 9.77 μg/mL, or 4.88 μg/mL to the ND-vaccinated birds, except for the vaccine control (VC), where *n* = 30 per group. *In vivo* results show that GPS-1 combined with Newcastle disease (ND) vaccine had the best efficacy at significantly increasing chickens' body weight and ND serum antibody titer, enhancing their secretion of IL-2 and IFN- γ, and promoting the development of immune organs. The results also indicate that GPS-1 was able increase the proliferation of *in vitro* immune cells and elevate their cytokine secretion, which enhances the body's immune response. GPS-1 also clearly has the potential to be used as an immune adjuvant alongside ND vaccination.

## Introduction

Over the past few decades, researchers have isolated various polysaccharides from plants, animals, and microorganisms, and have demonstrated their effective therapeutic activities and relatively low toxicities to mammals. As a result, there is increasing attention to polysaccharides in the field of molecular therapeutics. Prior studies have demonstrated that water-soluble polysaccharides extracted from Chinese herbal medicines provide medicinal benefits such as immune regulation, antioxidation, antitumor activity, liver protection, and anticoagulation (1–3).

Licorice has been used for over 2,000 years in traditional Chinese medicine to treat many human ailments like peptic ulcers, Addison's disease, asthma, cough, sore throat, acne, and boils (4–6). Chinese licorice is still a commonly used medicine in many parts of Asia. It comes from the dried roots and rhizomes of Glycyrrhiza uralensis, and is mainly found in northwest China, including the regions of Xinjiang, Gansu, and Shanxi. Its main active components are triterpenoid saponins and flavonoids, which endue Glycyrrhiza uralensis with potential pharmacological applications as an anti–ulcer, anti–inflammatory, anti–spasmolysis, antioxidant, antiviral, antidepressant, liver protectant, expectorant, and memory enhancement compound (7–10). There is a growing body of evidence that the polysaccharides within many plants like Glycyrrhiza uralensis may prove useful with immunomodulation, lowering glucose, or as a novel antiviral or antitumor agent (11–17). Polysaccharides are a type of sugar moiety with complex molecular structures, created by the condensation and dehydration of several monosaccharide molecules. Glycyrrhiza polysaccharides (GPS) are the main active macromolecules in Glycyrrhiza uralensis (18, 19), and have previously shown promising results as an antibacterial (20), antiviral and antitumor agent (21, 22), a liver protectant (23), and immune regulator (14). In one study that highlighted this immune-regulation potential, Glycyrrhiza polysaccharide was fed to CT-26 tumor-bearing mice and significantly inhibited tumor growth, increased the immune organ index, activated CD4+ and CD8+ immune cells, and increased levels of IL-2, IL-6, and IL-7 cytokines (24). Because many other polysaccharides have been reported have both immunomodulatory and antioxidant effects (25–28), the antioxidant properties of GPS-1 were tested in the present research. In a separate study, Glycyrrhiza polysaccharide was extracted by supercritical CO_2_, and an antioxidation experiment was carried out *in vitro* (29). In that investigation Glycyrrhiza polysaccharide was found to possess a notable ability to scavenge OH, O_2_, and DPPH. Recently, many studies have suggested that Glycyrrhiza polysaccharides perform a wide range of biological functions. Research reported that dietary supplementation with Glycyrrhiza polysaccharide improves the growth, development, and serum antioxidant levels of chickens raised for meat (30). In addition, Glycyrrhiza polysaccharides also has the ability to improve growth, and the quantities of white blood cell, neutrophils, red blood cells, and platelets, as well as elevate alkaline phosphatase, total protein, globulin, glucose, triglycerides, immunoglobulin A, immunoglobulin G, total antioxidant capacity levels, and immune responses in mice, quails, and weaned piglets (31–33). In this study, we investigated the immune enhancement and antioxidation functions of GPS-1 *in vitro* and *in vivo* (intramuscular injection, unlike previous studies on oral GPS-1). Our aim is to contribute to a deeper understanding of the immunoenhancement and antioxidant activities of GPS-1, and to provide a theoretical basis for its continued development and application as a pharmaceutical.

## Materials and methods

### Materials and chemicals for *in vitro* experiments

GPS-1 was prepared following previously reported methods (14). The crude polysaccharide was extracted from licorice by the Sevag method, deproteinated by DEAE chromatography column, purified by Sepharose CL−6B and Sephadex G-200 chromatography columns, and lyophilized to get purified GPS-1 extract. RPMI 1640 and DMEM were purchased from GIBCO. The penicillin-streptomycin mixture was sourced from HyClone. Fetal bovine serum was purchased from Tianhang Biotechnology Co., Ltd. Lohmann Brown chickens' recombinant proteins GM-CSF (mGM-CSF) and interleukin 4 (IL-4) were purchased from Peprotech Inc. The MTT solution and heparin was bought from Biofroxx. Phytin (PHA-P) was procured from Solarbio. Human peripheral blood lymphocytes separation medium was purchased from Tianjin Haoyang Biological Products Technology Co., Ltd. Histopak separators were bought from Sigma. IL-1β, Il-12, IL-12P70, IFN-γ, TNF-α, and NO kits were purchased from Nanjing Aoqing Biotechnology Co., Ltd. DPPH was obtained from TCI Chemical Industrial Development Co., Ltd RAW 264.7 cells were purchased from ATCC. ABTS and tromethamine were purchased from Soleil.

### Safe concentration of GPS-1 for peripheral lymphocytes

First, the feathers were removed from the blood collection site on the breast and the skin was sterilized with 75% alcohol. Then, a sterile syringe was inserted into the heart to collect 5 mL of blood and immediately mixed with heparin sodium. The fresh blood with anticoagulant was mixed with isotonic saline in a ratio of 1:1. Then, human peripheral blood lymphocyte separation medium was added to the top layer of lymphocytes and centrifuged at 1,500 rpm for 15 min. The second layer of lymphocytes was collected and put into a test tube containing isotonic saline. These lymphocytes in saline were mixed and centrifuged again at 1,500 rpm for 20 min. This procedure was repeated once more to obtain peripheral blood lymphocytes. The lymphocytes were then resuspended to 1 × 10^6^ cells/mL with RPMI 1,640 medium containing 10% fetal bovine serum and 1% penicillin-streptomycin, then cultured in 96-well-culture plates at 100 μL per well (34).

A series of GPS-1 concentrations (2.44, 4.88, 9.77, 19.53, 39.06, 78.12, 156.5, 312.5, 625, and 1,259 μg/mL) were added to 96-well-culture plates at 100 μL per well, with 4 wells per concentration. The 96-well-plates were then cultured in a humidified incubator at 37°C and 5% CO_2_ for 44 h. Then MTT solution (5 mg/mL, 30 μL/well) was added, and incubation was continued for 4 more h. Afterward, 100 μL DMSO per well was used to dissolve the precipitates while shaking for 5 min. An enzyme-linked immunosorbent assay (ELISA) reader was then used to analyze the absorbance of the solution at a wavelength of 570 nm. At the GPS-1 concentration of 39.06 μg/mL the A570 value is significantly higher than the lymphocyte control group. 39.06 μg/mL was therefore chosen as the maximum safe concentration of GPS-1.

### Immune cell proliferation assays

The peripheral blood lymphocytes were prepared according to methods detailed in section Immune cell proliferation assays. The spleen cell suspension was filtered with a 200 mesh cell filter and separated using lymphocyte separation solution. After centrifugation at 2,000 rpm for 10 min, the middle layer containing lymphocytes was isolated and washed twice with PBS to remove impurities. More than 95% of spleen lymphocytes were viable based on trypan blue dye exclusion.

The immature Lohmann Brown chickens bone marrow dendritic cells (chBM-DCs) were obtained from 4-week-old Lohmann Brown chickens following our previously reported method (35). The femurs and tibias were removed and separated from the surrounding muscle tissue under aseptic conditions. Then, the bones were washed three times with 0.01 M PBS (pH 7.2). Clusters of bone containing marrow were collected and centrifuged at 1,500 rpm for 20 min to obtain the precipitate. This precipitate was disaggregated in PBS and centrifuged at 1,500 rpm for 20 min to remove the supernatant solution. The cells were resuspended in PBS, layered onto an equal volume of Histopaque®-1077 solution (Sigma-Aldrich) and centrifuged at 2,500 rpm for 30 min. The immature chBM-DCs cells at the interface were then collected and suspended in complete DMEM cell culture medium (Sigma) containing 10% fetal bovine serum (FBS) and 1% penicillin-streptomycin (p/s).

The peripheral blood lymphocytes and chBM-DCs were each adjusted to 5.0 × 10^6^ cells/mL and added into separate 96-well-cell culture plates at 100 μL per well. Based on our determination of the safe concentration of GPS-1 from section Immune cell proliferation assays, GPS-1 was added at doses of 2.44, 4.88, 9.77, 19.53, and 39.06 μg/mL, with four wells per concentration. The cultures control (CC) group and PHA control were also cultured accordingly. All 96-well-plates were then incubated at 37°C and 5% CO_2_ for 44 h. After cultivation, the proliferation rate of each well was tested *via* MTT assay following the method in Immune cell proliferation assays.

### Supernatant cytokines and NO assay

#### Supernatant cytokines of peripheral lymphocytes assay

The peripheral blood lymphocytes were cultured in 24-well-cell culture plates with a cell concentration of 1 × 10^6^ cells/mL following a previously published method (36). GPS-1 was added at a range of non-toxic concentrations (2.44, 4.88, 9.77, 19.53, and 39.06 μg/mL) to each cell culture plate, each in triplicate, and incubated at 37°C and 5% CO_2_ for 48 h. The culture control (CC) group and PHA control were also incubated with the same conditions (*n* = 3), minus GPS-1 treatment. Then, the supernatants of each group were collected. The concentrations of IL-12, IFN-γ, and TNF-α in the supernatants of each group were measured using ELISA kits.

#### Supernatant cytokines and NO production of ChBM-DCs precursor cells

The chBM-DC precursor cells were harvested and cultured in 24-well-plates at 37°C and 5% CO_2_ for 15 h. After discarding the suspended cells, the adherent cells were incubated in the DMEM complete media (10% FBS, 1% p/s) containing GM-CSF (50 ng/mL) and IL-4 (50 ng/mL) stimulators. On the 3rd and 5th days of incubation, half of the nutrient solution was refreshed. On the 6th day of cultivation, GPS-1 of different concentrations (2.44, 4.88, 9.77, 19.53, and 39.06 μg/mL) was added to all but the CC and LPS groups, then cells were cultivated for another 48 h. The treated cells were divided into two parts. The supernatant of each well was used to measure the concentration of secreted NO in each group (using Griess reagent), and quantify the production of cytokines IL-2, IL-1β, IFN-γ, TNF-α, and IL-12p70 using ELISA kits (37).

### Antioxidant activity of GPS-1 *in vitro*

#### Hydroxyl radical scavenging activity assay

Hydroxyl radical scavenging activity was examined using the salicylic acid trapping method (38). In brief, 2 mL of FeSO_4_ solution (9 mmol/L), 2 mL of salicylic acid solution (9 mmol/L), and 2 mL of GPS-1 (2.44, 4.88, 9.77, 19.53, or 39.06 μg/mL) were added to each test tube. Then, 2 mL of H_2_O_2_ solution (8.8 mmol/L) was added to initiate the reaction, which was kept at 37°C in a water bath for 30 min. The absorbance (A1) of each sample was measured at 510 nm. Ascorbic acid was used as a positive control while distilled water was used as blank control (A0). Each group was repeated in triplicate. The scavenging rate (%) was calculated according to the following equation:


$$\begin{array}{l} \text{Scavenging rate}\left(\text{\%}\right)=\left[1-\frac{\text{A}1}{\text{A}0}\right]\times 100\text{\% } \end{array}$$

#### DPPH radical scavenging assay

The DPPH radical scavenging assay was performed according to the previously published description (39). Briefly, 1 mL of DPPH-ethanol solution (1 × 10^−4^ mol/L) was mixed with 3 mL of each concentration of GPS-1 (2.44, 4.88, 9.77, 19.53, and 39.06 μg/mL), and kept in darkness at 37°C for 30 min. Absorbance (A1) was measured at 517 nm. Ascorbic acid was used as positive control, distilled water was the blank control (A0), and ethanol was used to determine A2. Each group was tested in triplicate. The DPPH radical scavenging effect (%) was calculated as follows:


$$\begin{array}{l} \text{DPPH Scavenging rate }\left(\text{\%}\right)=\left[1-\frac{\text{A}1-\text{A}2}{\text{A}0}\right]\times 100\text{\% } \end{array}$$

#### ABTS radical scavenging activity assay

ABTS radical scavenging activity was measured according to Roberta Bernini's method (29), with minor modifications. The ABTS radical solution mixture (7 mmol/L of ABTS solution and 2.5 mM of potassium persulfate, 1:1, v/v) was diluted with ethanol to an absorbance (A0) of 0.70 ± 0.02 at 734 nm. Then, 0.2 mL of GPS-1 at different concentrations (2.44, 4.88, 9.77, 19.53, and 39.06 μg/mL) was combined with 0.8 mL of ABTS radical solution mixture, and allowed to react for 6 min in darkness, at room temperature. The absorbance (A1) of each product was measure at 734 nm. BHT was used as the positive control. The ABTS radical scavenging activity of GPS-1 was calculated with the following equation:


$$\begin{array}{l} \text{ABTS radical scavenging activity }\left(\text{\%}\right)=\left[1-\frac{\text{A}1}{\text{A}0}\right]\times 100\text{\% } \end{array}$$

#### Superoxide anion radical scavenging ability assay

Superoxide anion radical scavenging ability was assayed according to a previously reported method (35, 40). 2 mL of tris-HCl buffer solution (0.05 mol/L, pH 8.2) was mixed with each GSP-1 concentration (2.44, 4.88, 9.77, 19.53, and 39.06 μg/L), 1 mL each. These mixtures were combined with pyrogallol solution (7 mmol/L, 0.6 mL) and kept in a 25°C water bath for 30 min then combined immediately afterward. The absorbance of the combination was detected at 320 nm every 30 s and labeled “A1.” The absorbance of the blank control group (A0) was determined using pyrogallol solution (7 mmol/L, 2 mL). The superoxide anion clearance can be calculated as follows:


$$\begin{array}{l} \text{Superoxide anion clearance }\left(\text{\%}\right)=\left[1-\frac{\text{A}1}{\text{A}0}\right]\times 100\text{\% } \end{array}$$

#### Cell viability assays

RAW 264.7 cells were used to measure cell survival after GPS-1 treatment. Cell density was adjusted to 1 × 10^6^ cells/mL and added into three 96-well-plates at 100 μL per well (41–43).

The first plate was cultured for 30 min at 37°C and 5% CO_2_. H_2_O_2_ was added to all wells except the control group at a range of concentrations (100, 200, 300, 400, 500, 600, 700, 800, 900, 1,000 μM), each in triplicate. The plate was then incubated for another 24 h. Afterward, 30 μL of MTT solution was added to each well, followed by another 4 h of incubation. An ELISA reader was then used to detect absorbances at a 570 nm, allowing us to quantify the damage index of H_2_O_2_. Each concentration was repeated in triplicate.

A second 96-well-plate seeded with RAW 264.7 cells and 0.1 mL of each GPS-1 dose (2.44, 4.88, 9.77, 19.53, 39.06, 78.12, and 156.50 μg/L) was added (*n* = 3 per group). The plate was incubated at 37°C and 5% CO_2_ for 24 h. The MTT solution (30 μL) was added into each well, then incubated for another 4 h. After cultivation, the supernatants were discarded, then the precipitates were dissolved in 100 μL DMSO and shaken for 15 min. The absorbance of the solution in each well was detected using an ELISA reader at a wavelength of 570 nm to elucidate the stimulative effects of GPS-1.

Similarly, the third 96-well-plate was treated with different concentrations of GPS-1 (4.88, 9.77 and 19.53 μg/L) and incubated for 24 h. The control group, H_2_O_2_ model groups, and positive control (25 μg/ml vitamin C) group were also incubated for 24 h. Except for the control group, each group was also treated with 900 μM H_2_O_2_ for 4 h. An MTT assay was used to analyze the ability of GPS-1 to protect RAW 264.7 cells from oxidative damage due to H_2_O_2_ exposure.

### Materials and chemicals for *in vivo* experiments

The source of GPS-1 is detailed in section Materials and chemicals for *in vitro* experiments, above. RPMI 1640 and DMEM were purchased from GIBCO. Newcastle disease IV vaccine (La Sota Strain) was purchased from Nanjing Qian Yuan Hao Biological Co., Ltd. IL-2, IL-4, and IFN-γ kits and T-AOC, GSH-Px, CAT, Sod and MDA kits were purchased from Nanjing Genscript Biotechnology Co., Ltd. The sodium chloride for injection was purchased from Chenxin Pharmaceutical Co., Ltd.

### Animals and conditions

One hundred and fifty unvaccinated, healthy, 1-day-old, male, Lohmann Brown chickens were purchased from Huang Mu Qiao Hatchery in Nanjing Luhe District, China and were housed at the Laboratory Animal Center of Nanjing Agricultural University. Chickens were given a standard diet and reared in standard specific pathogen free (SPF) conditions between 20 and 23°C, with 12–h light/dark cycles. Animal experiments were performed in compliance with the guidelines of the Nanjing Agricultural University Institutional Animal Care and Use Committee (IACUC), detailed in the IACUC-approved protocol (No.:2019BAD22B01).

#### Animal grouping and vaccination

One hundred and fifty unvaccinated, healthy, 1-day-old, male, Lohmann Brown chickens were randomly divided into five groups: GPS-1 low dose (GPSL) group (1 mg/mL), GPS-1 medium dose (GPSM) group (2 mg/mL), GPS-1 high dose (GPSH) group (4 mg/mL), VC group, and the unvaccinated BC group, with 30 per group. At 13-days-old, all of the Lohmann Brown chickens (except those in the unvaccinated BC group) were immunized with NDV-attenuated vaccine (La Sota; 170902) by intraocular-nasal vaccination, with a booster vaccination at 28 days of age. Concurrently with each vaccination, the three GPS-1 medicated groups were injected intramuscularly with 1 mL of GPS-1 at either low, medium, or high dosage for three consecutive days, while the VC group and BC group were treated with the same amount of saline (1 mL), respectively (44).

#### Serum antibody titer assays

Blood samples were collected from the brachial vein on days 7 (D7), 14 (D14), 21 (D21), 28 (D28), and 35 (D35), after the first vaccination. Serum was tested for antibodies against Newcastle disease (ND) using the HA-HI method, while the IL-2 and IFN-γ levels in serum were determined using ELISA kits.

#### Morphological changes of immune organs analysis

On the 14th day (D14) after vaccination, the spleen, bursa of Fabricius, and thymus were harvested and fixed in 4% paraformaldehyde solution for 24 h. These immune organs were H&E stained and their morphological changes scrutinized under a light microscope.

### Data analysis

Statistical data were analyzed by SPSS 19.0 software and the results were described as the mean ± SE. The least significant difference test was used to determine the significant difference of each mean; *P* < 0.05 was considered statistically significant.

## Results

### Effects of GPS-1 on immune cell proliferation *in vitro*

As shown in Table 1, there is no significant difference between the A570 values of the 625 μg/mL GPS-1 group and the cell control group. Thus, 625 μg/mL was determined to be the maximum safe concentration. However, the A570 value of the 39.06 μg/mL GPS-1 group was significantly higher than that of the cell control group (*P* < 0.05), so 2.44–39.06 μg/mL were chosen as the concentrations of GPS-1 in cell proliferation tests for the present investigation.

**Table 1:** A570 value of every polysaccharide groups 1,250–2.44 μg mL^−1^ (*n* = 4).

**Concentration**	**A_570_**
1,250	0.203 ± 0.010^e^
625	0.347 ± 0.067^d^
312.5	0.356 ± 0.010^d^
156.5	0.355 ± 0.014^d^
78.12	0.386 ± 0.005^bc^
39.06	0.409 ± 0.013^ab^
19.53	0.418 ± 0.017^a^
9.77	0.398 ± 0.038^ab^
4.88	0.369 ± 0.007^cd^
2.44	0.344 ± 0.017^d^
CC	0.365 ± 0.005^cd^

a−e Bars in the histogram with no identical letters indicated a significantly difference (*P* < 0.05). CC (cell control), the same below.

#### GPS-1 increased lymphocyte proliferation

**Figure 1:**
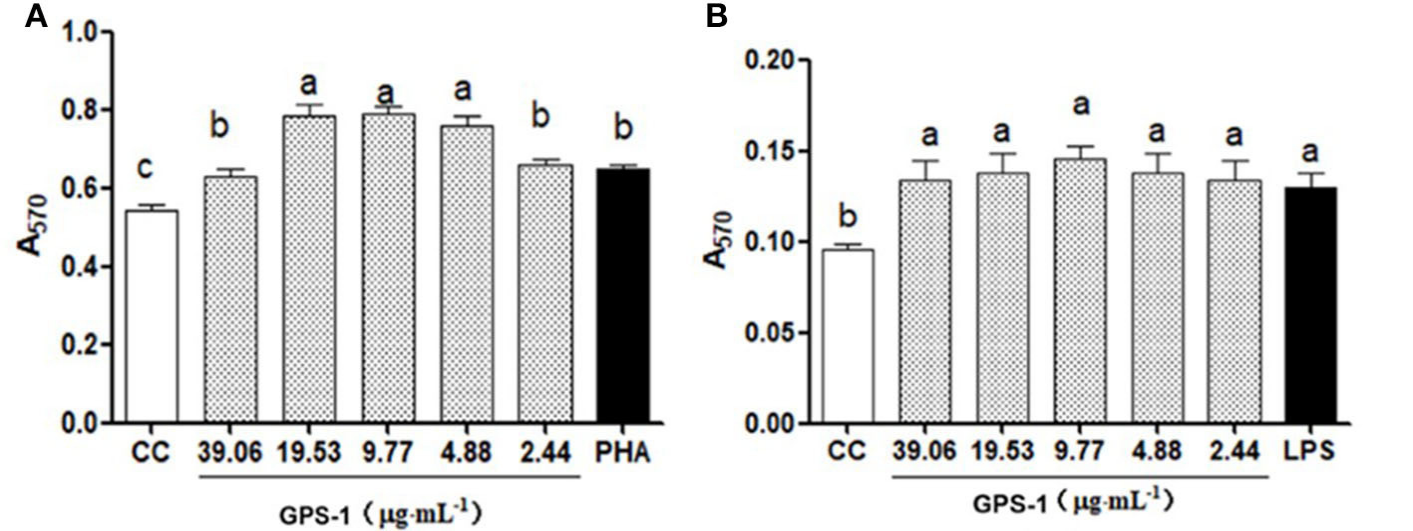
Effects of GPS-1 on A570 of chicken peripheral **(A)** and spleen lymphocytes **(B)**. ^a−c^Values in the same column with different superscript letters differ significantly at *p* < 0.05.

In this experiment, MTT assays were used to detect the effects of GPS-1 on Lohmann Brown chickens' peripheral blood lymphocytes and splenic lymphocytes *in vitro*. The results in Figure 1 show that GPS-1 at doses of 4.88, 9.77, 19.53, and 39.06 μg/mL can significantly promoted the proliferation of peripheral blood lymphocytes, compared with the phytohemagglutinin (PHA) control group (*P* < 0.05). Within the safe concentration range, GPS-1 could also significantly promote the proliferation of splenic lymphocytes, but there were no significant differences between the doses of GPS-1 and cell control (CC) groups (*P* > 0.05).

#### GPS-1 induces chBM-DC precursor cell proliferation

**Figure 2:**
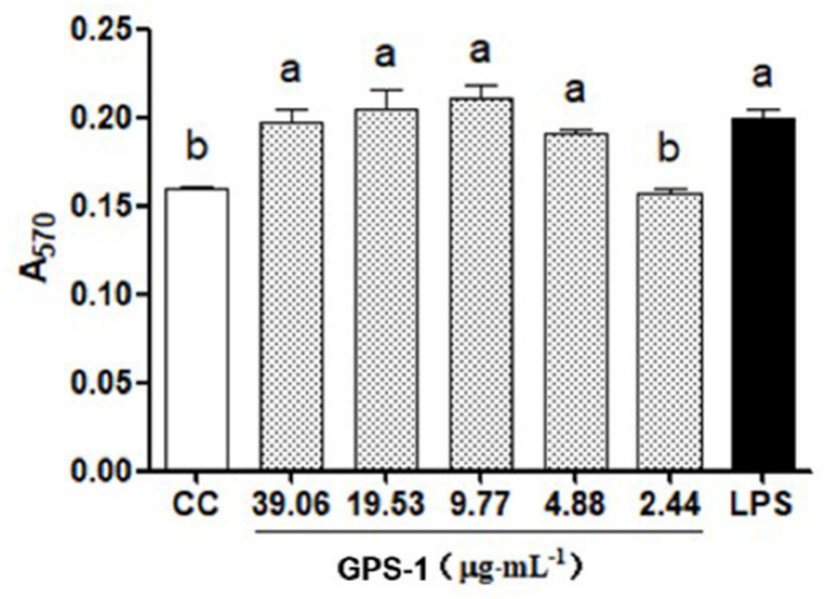
GPS-1 effects on the proliferation of chicken dendritic cells (A570). ^a, b^Values in the same column with different superscript letters differ significantly at *p* < 0.05.

The results in Figure 2 show that GPS-1 in the range of 4.88 to 39.06 μg/mL can significantly promote the proliferation of chBM-DCs progenitor cells (*P* < 0.05), but there was no significant difference between the control group and the concentration of 2.44 μg/mL GPS-1 (*P* > 0.05). Thus, the range of concentrations that can effectively enhance the activity of the chBM-DCs progenitor cells is 4.88–39.06 μg/mL.

### Effect of GPS-1 on the secretion of cytokines by immune cells

#### Lymphocyte expression analysis

**Figure 3:**
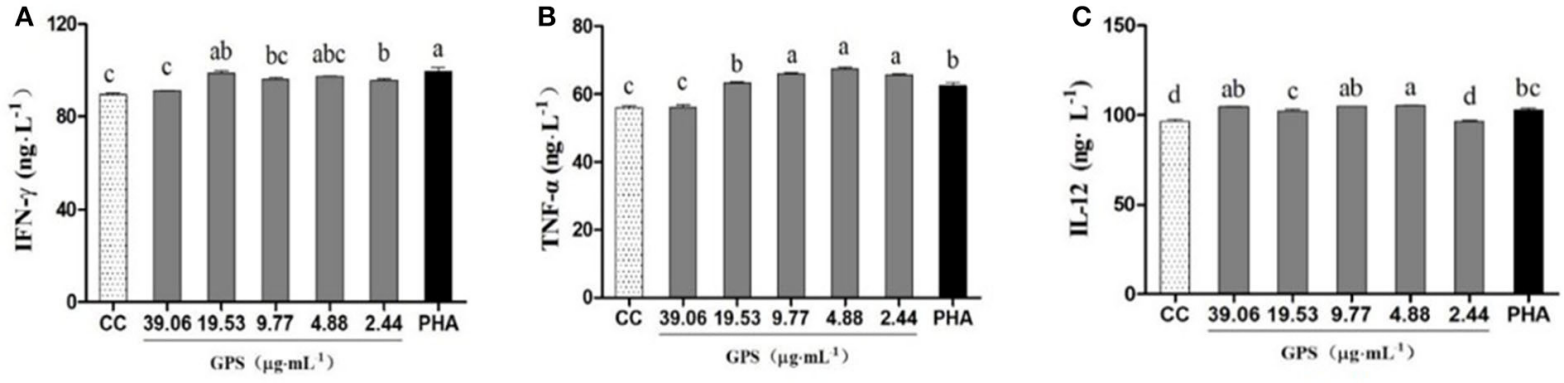
Effects of GPS-1 on the secretion of cytokines by lymphocytes. **(A)** IFN-γ; **(B)** TNF-α; **(C)** IL-12. ^a−d^Values in the same column with different superscript letters differ significantly at *p* < 0.05.

Data in Figure 3 indicates the levels of IFN-γ and TNF-α were significantly higher in groups treated with GPS-1 doses from 2.44 to 19.53 μg/mL than in the cell control group (*P* < 0.05). Groups given GPS-1 at concentrations of 4.88–39.06 μg/mL also had substantially elevated IL-12, vs. the cell control (CC) group. The most effective GPS-1 dose was 4.88 μg/mL, in which IL-12 was significantly higher than the PHA control group (*P* < 0.05).

#### chBM-DCs expression analysis

**Figure 4:**
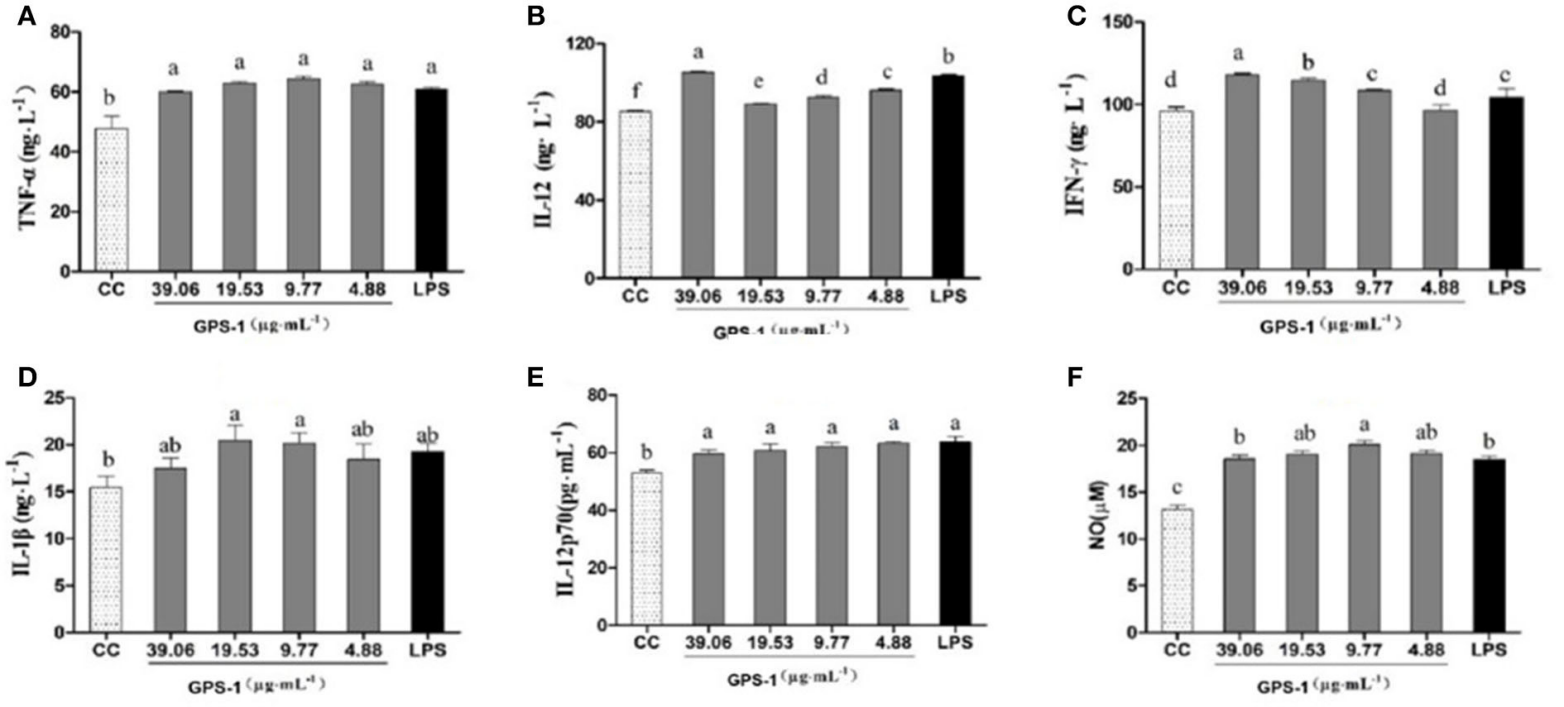
Effects of GPS-1 on the secretion of NO **(F)**, and cytokines **(A–E)** by DCs. **(A)** TNF-α; **(B)** IL-12; **(C)** IFN-γ; **(D)** IL-1β; **(E)** IL-12p70; **(F)** NO. ^a−f^Values in the same column with different superscript letters differ significantly at *p* < 0.05.

The effects of GPS-1 on the secretion of TNF-α, IL-12, IFN-γ, IL-1 β, and IL-12p70 by chBM-DCs are shown in Figure 4. The secretion of TNF-α (Figure 4A) was significantly higher in groups treated with GPS-1 at concentration of 4.88–39.06 μg/mL than in the CC group (*P* < 0.05), but compare with the LPS group there was not a significant difference (*P* > 0.05). GPS-1 groups in the range of safe concentrations exhibited extremely elevated IL-12 secretion (Figure 4B), a significant improvement over the CC group (*P* < 0.05). Moreover, the IL-12 level in the 39.06 μg/mL GPS-1 group was significantly higher than in the LPS control group. As shown in Figure 4C, the IFN-γ secretion of groups treated with 9.77–39.06 μg/mL GPS-1 was higher than that of the CC group and the difference is significant(*P* < 0.05). Additionally, GPS-1 groups at concentrations of 19.53 μg/mL and 39.06 μg/mL have higher IFN-γ secretion than the LPS group and the difference is significant (*P* < 0.05). Compared with the CC group, the secretion of IL-1β (Figure 4D) was higher in GPS-1 groups at the concentrations of 9.77 μg/mL and 19.53 μg/mL (*P* < 0.05). However, the remaining GPS-1 groups and the LPS group showed no significant difference with the CC group (*P* > 0.05). The IL-12p70 secretion (Figure 4E) of all GPS-1 groups and the LPS group was significantly higher than that of the CC group (*P* < 0.05). Moreover, compared with the CC group, GPS-1 at 9.77 μg/mL significantly increased the quantity of NO (Figure 4F) expelled by DCs (*P* < 0.05).

The present results showed that the NO content of endocrine DCs was significantly higher at safe concentrations of GPS-1(4.88, 9.77, 19.53, and 39.06 μg/L) than in the CC control group (Figure 4F). Additionally, the GPS-1 concentration of 9.77 μg/mL produced significantly higher NO than the LPS group. The results indicate that GPS-1 can significantly promote the secretion of NO by dendritic cells and further enhance their immune function.

### Antioxidant activity

#### Hydroxyl radical scavenging activity

**Figure 5:**
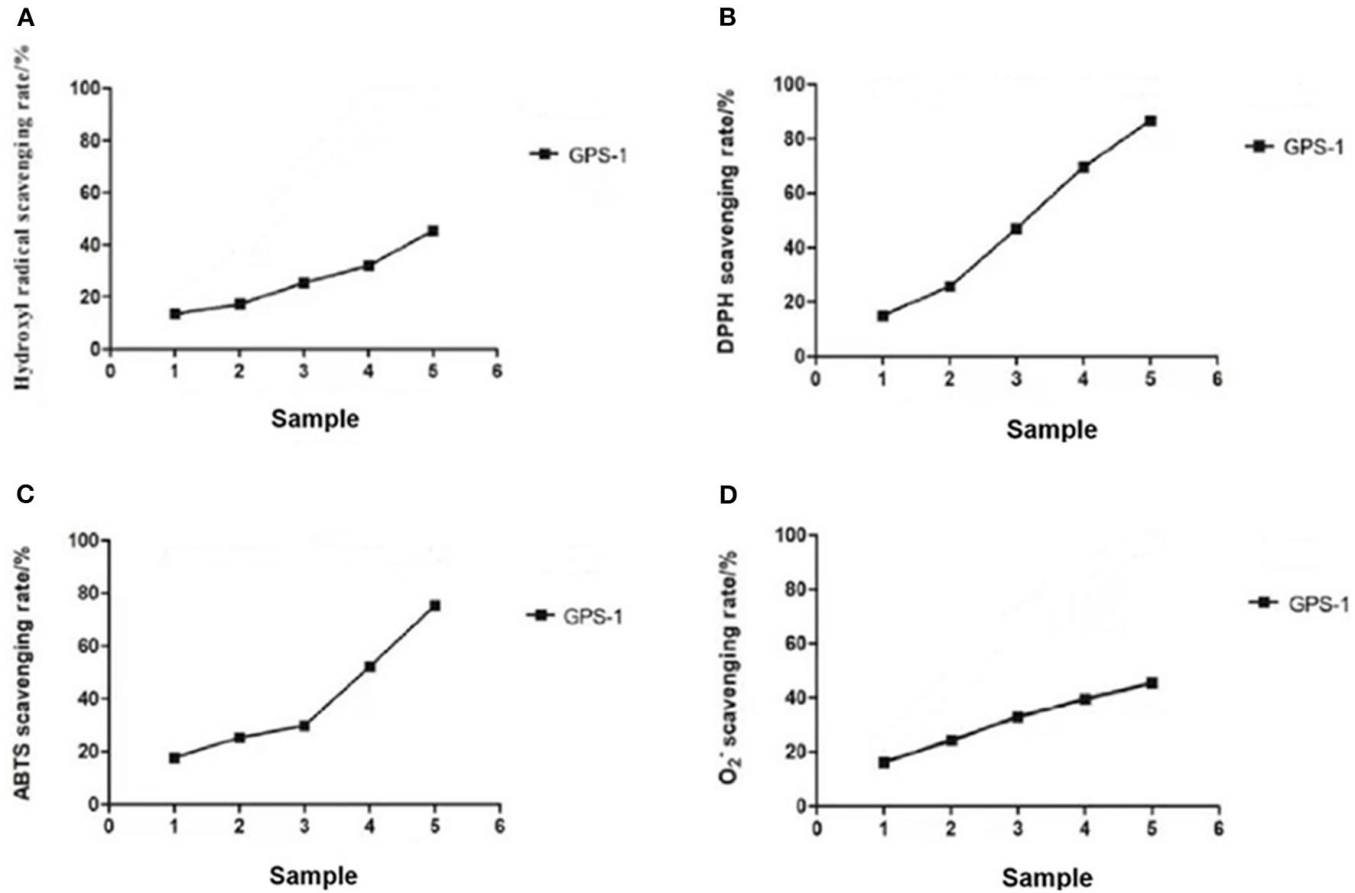
The hydroxyl radical-removing rate (%) **(A)** The DPPH radical-removing rate (%) **(B)** The ABTS radical-removing rate (%) **(C)** Superoxide anion radical-scavenging rate (%) **(D)** Sample numbers 1–5 correspond to the concentration of GPS-1 2.44, 4.88, 9.77, 19.53, and 39.06 μg/mL, respectively.

In Figure 5A, note the uptick in antioxidant capacity with the corresponding increase in GPS-1 concentration in an apparent dose-dependent relationship. At GPS-1 concentrations between 1 and 5 mg/mL, the scavenging rate rose from 13.47 to 45.29%. Meanwhile, the scavenging rate of ascorbic acid, the positive control (VC), increased from 16.55% at 1 mg/mL to 96.82% at 4.0 mg/mL, where it plateaued. These results show that GPS-1 can scavenge hydroxyl radicals, but is much less effective than ascorbic acid.

#### DPPH radical scavenging activity

Figure 5B summarizes the scavenging activities of GPS-1. VC (the positive control) exhibited 98.39% antioxidant activity at 1 mg/mL, then plateaued. The antioxidant activity of GPS-1 increased with concentration in a dose-dependent manner. The scavenging rate developed from 15.06% at a GPS-1 concentration of 1 mg/mL to 86.60% at 5 mg/mL, and met its half maximal inhibitory concentration (IC50) at 3.07 mg/mL GPS-1.

#### ABTS radical scavenging activity

The scavenging effects of GPS-1 and VC on ABTS radicals are examined in Figure 5C. Higher concentrations of GPS-1 improved scavenging activity significantly. The scavenging ratio of GPS-1 rose slowly between 1 and 3 mg/mL, then rose sharply between 3 and 5 mg/mL. The IC50 value of GPS-1 was 3.7 mg/mL. Likewise, VC displayed an excellent ability to scavenge ABTS radicals at 93.53–99.42% at concentrations ranging from 1 to 5 mg/mL.

#### Superoxide anion radical scavenging ability

The results of the superoxide anion radical scavenging experiment are given in Figure 5D. The antioxidant capacities of GPS-1 and ascorbic acid are both in dose-dependent, linear relationships with their concentrations. The scavenging rate of GPS-1 increased from 16.21% at a GPS-1 concentration of 1 mg/mL to 45.46% at 5 mg/mL, while the scavenging ability of VC grew from 22.87 to 97.58% within the same dosage range.

#### Effect of GPS-1 on RAW 264.7 cells

**Figure 6:**
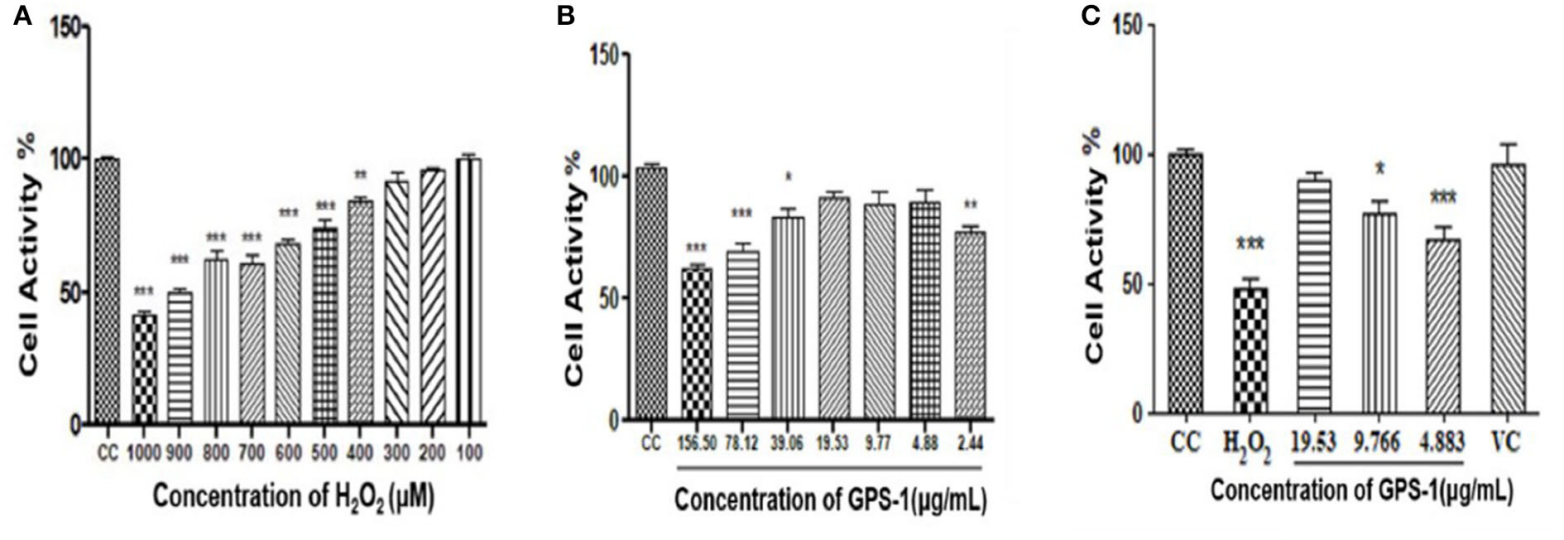
Effect of H_2_O_2_ on the survival rate of RAW 264.7 cells **(A)** Effect of GPS-1 on RAW 264.7 proliferation **(B)** Protective effect of GPS-1 against oxidative damage to RAW 264.7 cells induced by H_2_O_2_ **(C)** Protective effect of GPS-1 on RAW 264.7 cells exposed to H_2_O_2_. * stands for *P* < 0.05; ** stands for *P* < 0.01; *** stands for *P* < 0.001.

As shown in Figure 6A, the survival rate of RAW 264.7 cells declined with the increase of H_2_O_2_ concentration. When the concentrations of hydrogen peroxide reached 400 μM or above, cell proliferation was profoundly inhibited (*P* < 0.001). The lethality of hydrogen peroxide at 900 μM was 50.35%, which was selected as the treatment dose necessary to induce an oxidative damage model for RAW 264.7 cells.

As seen in Figure 6B, GPS-1 at 2.44 μg/mL and from 39.06 to 156.50 μg/mL significantly inhibited the growth of RAW 264.7 cells compared to the CC group (*P* < 0.05). Therefore, 4.883 μg/mL (GPSL), 9.77 μg/mL (GPSM) and 19.53 μg/mL (GPSH) were selected as the GPS-1 treatment concentrations.

The protective effect of GPS-1 on RAW 264.7 cells exposed to H_2_O_2_ can be observed in Figure 6C. After hydrogen peroxide treatment, both the high (GPSH,19.53 μg/mL) and middle (GPSM, 9.766 μg/mL) doses of GPS-1 showed significant protective effects on the RAW 264.7 cells exposed to H_2_O_2_, as compared to the control group (*P* < 0.05). Relative to the CC group, the cell activities of the H_2_O_2_ and GPSL decreased significantly. GPSHexhibited an excellent 89.84% cell survival rate after hydrogen peroxide damage. The activity of RAW 264.7 cells in the GPSM group (9.77 μg/mL) was only 76.93%, which is lower than that in the cell control (CC) group (*P* < 0.05). However, compared to the hydrogen peroxide group, the cell activity of the GPSM group was significantly increased (*P* < 0.01). The GPS-1 concentration of 4.883 μg/mL also increased cell viability relative to the H_2_O_2_ group, but not significantly.

### Immune enhancement effect of GPS-1 with Newcastle disease vaccine in Lohmann Brown chickens

#### Changes in serum antibody titer

**Figure 7:**
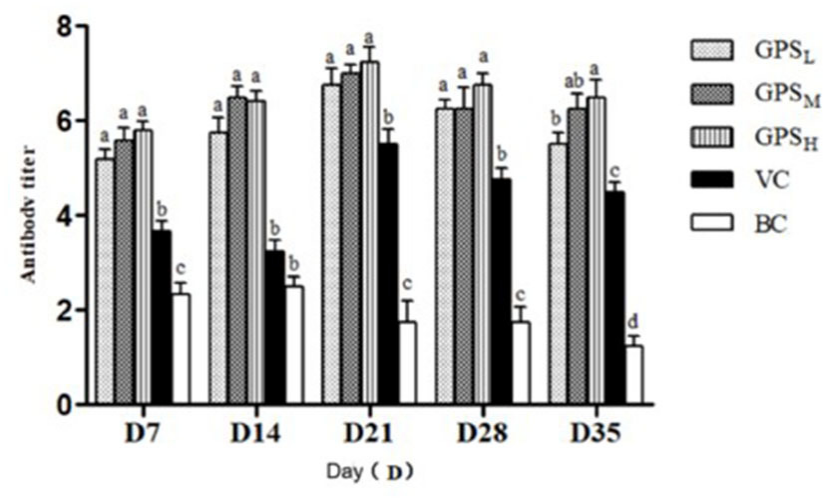
Changes in serum antibody titer (Log2). ^a−d^Values in the histogram with different letters indicate significant differences.

Variation in serum antibody titers between each group can be seen in Figure 7. On days 7, 21, and 35 after the first ND vaccination, antibody titers from GPS-1 treatment groups were significantly higher than those of VC and BC groups (*P* < 0.05). On days 14 and 28, the antibody titers of the GPSH and GPSM groups were also higher than these control groups (*P* < 0.05). Notably, the difference between the high and medium dose groups (GPSH and GPSM) was not significant (*P* > 0.05). Additionally, even in the lowest dose (GPSL) group the antibody titer was significantly higher than that of both control groups (*P* < 0.05).

#### The changes of serum IL-2 and IFN-γ concentration

**Figure 8:**
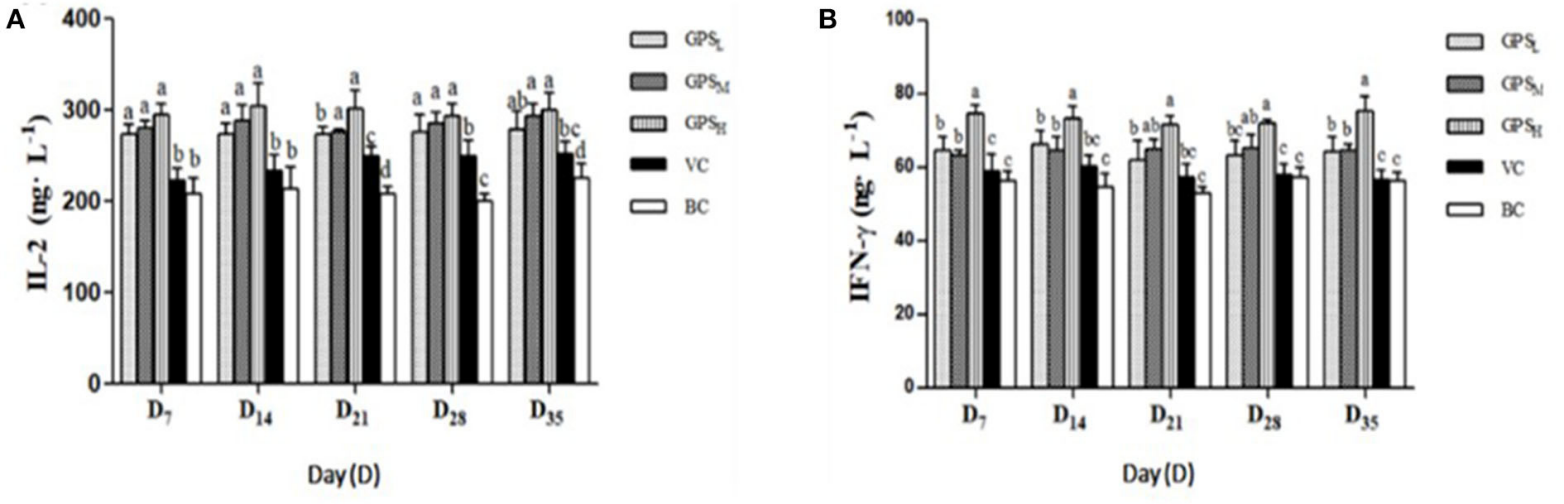
The changes of IL-2 **(A)** and **(B)** IFN-γ concentrations in each group (ng·L^−1^). ^a−d^Values in the histogram with different letters indicate significant differences.

According to Figure 8A, on days 7, 14, and 28 after vaccination, the IL-2 concentrations in the GPS-1 groups were higher than that in the VC and BC groups (*P* < 0.05), with the largest increase seen in the GPSH group. Similarly, on days 7, 14, and 35 the level of IFN-γ in each GPS-1 group was higher than in either the VC or BC groups (*P* < 0.05). Again, GPSH produced the largest response (*P* < 0.05). According to Figure 8B, on days 21 and 28, the IFN-γ content of the GPSH and GPSM groups was higher than that of the VC and BC control groups (*P* < 0.05).

#### Morphological changes of immune organs

**Figure 9:**
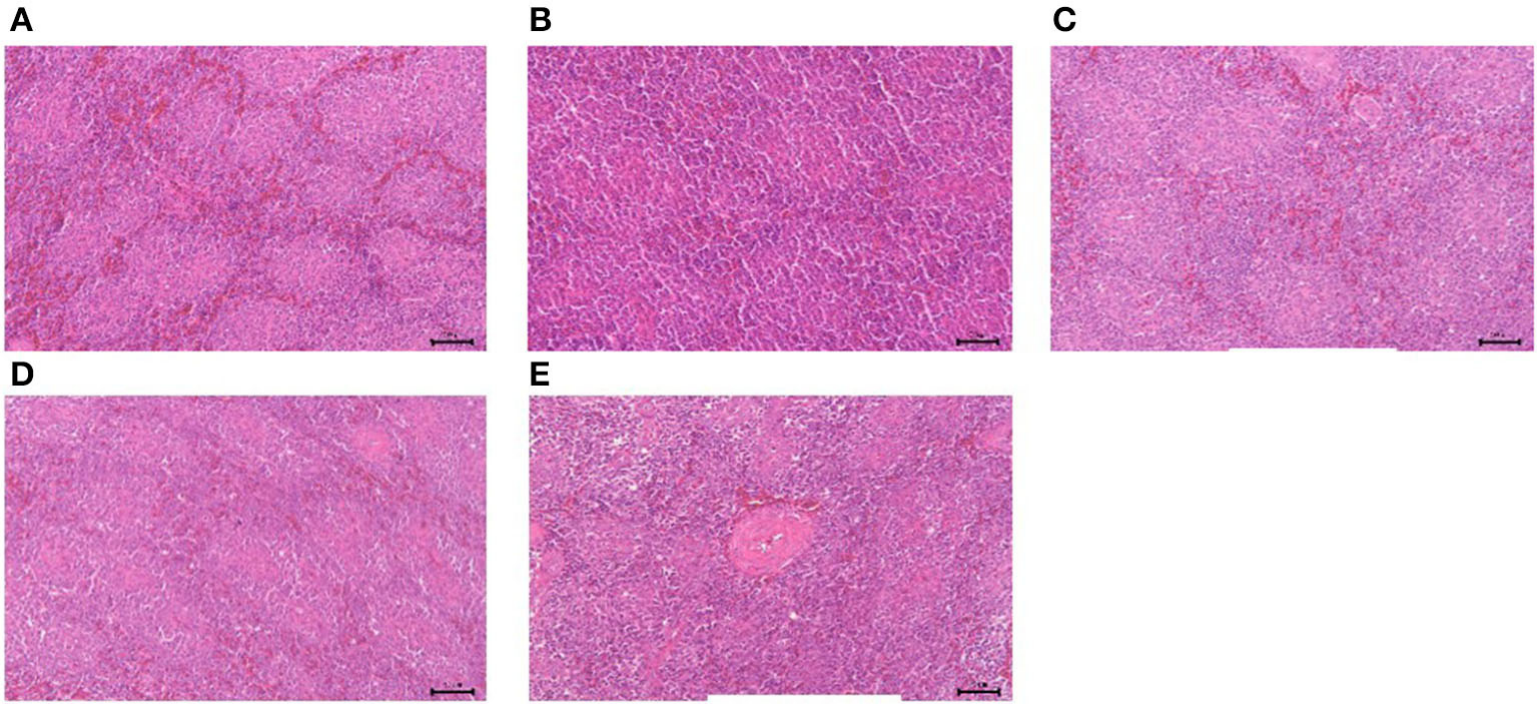
Histological changes of the spleen in each group. **(A)** GPS-1L group; **(B)** GPS-1M group; **(C)** GPS-1H group; **(D)** VC group; **(E)** BC group. H&E staining; 200× magnification.

**Figure 10:**
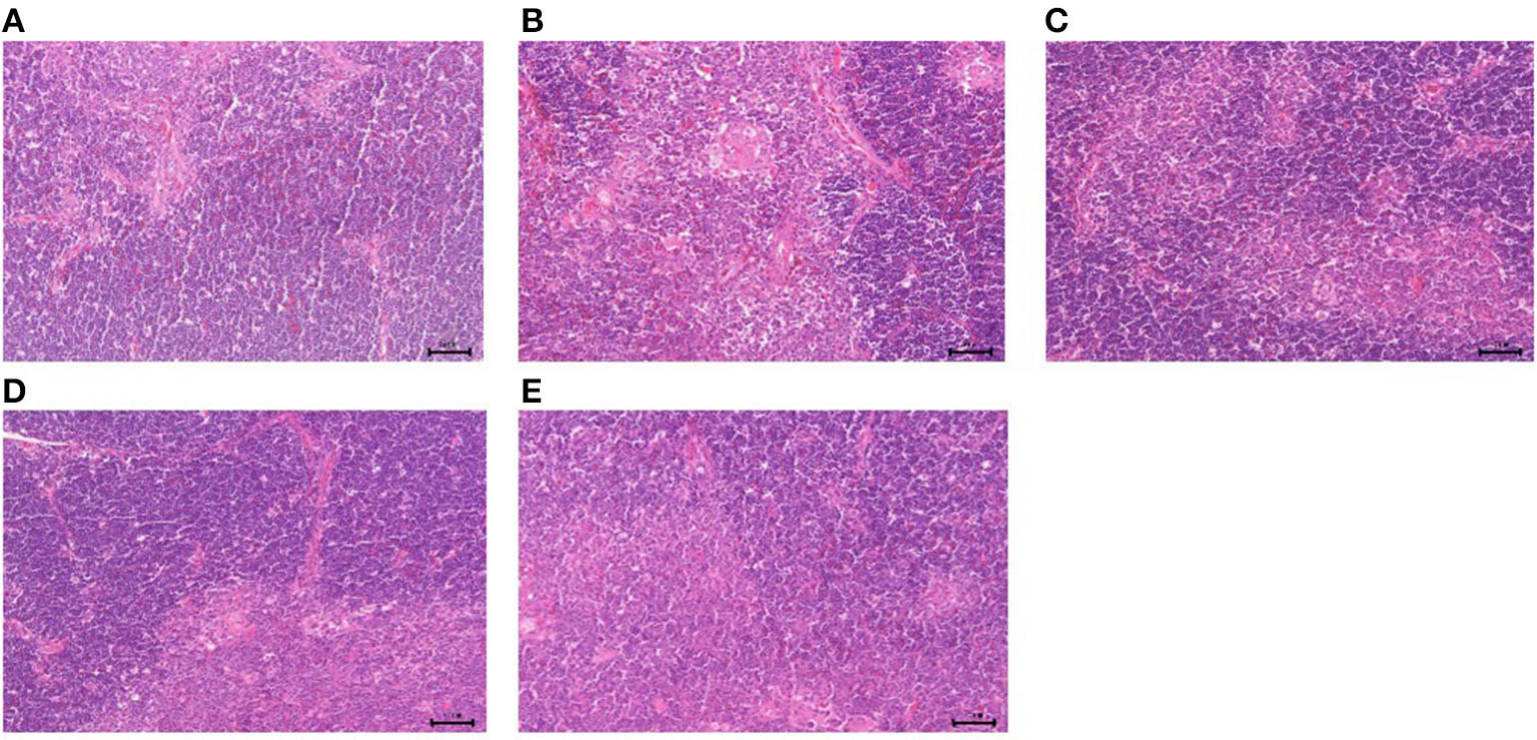
Histological changes of the thymus in each group. **(A)** GPS-1L group; **(B)** GPS-1M group; **(C)** GPS-1H group; **(D)** VC group; **(E)** BC group. H&E staining; 200× magnification.

**Figure 11:**
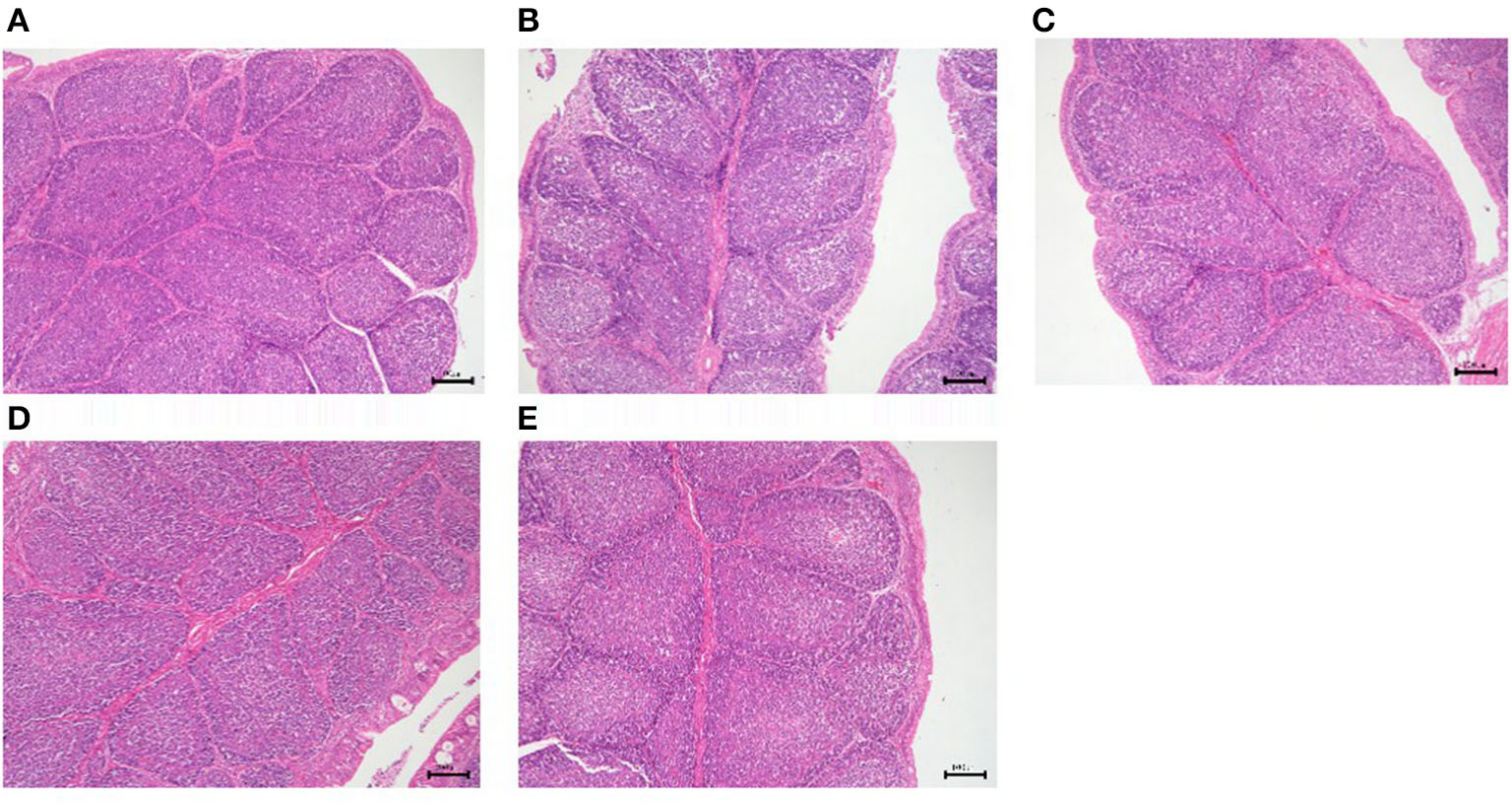
Histological changes of the bursa of Fabricius in each group. **(A)** GPS-1L group; **(B)** GPS-1M group; **(C)** GPS-1H group; **(D)** VC group; **(E)** BC group. H&E staining; 200× magnification.

The results of GPS-1 treatment on the spleen, thymus, and bursa of Fabricius of are presented in Figures 9–11, respectively. Compared with the VC and BC control groups, there was no observed toxic effect from GPS-1 on the spleen, thymus, or bursa of Fabricius.

## Discussion

The growth of chickens is affected by a variety of factors such as stocking density, temperature, pathogenic microorganisms, and immune performance, all of which affect the economic performance of poultry farmers and the prices consumers face in stores (45). Glycyrrhiza polysaccharide (GPS), the most abundant active component of Glycyrrhiza uralensis, has been the subject of a growing body of pharmacological and clinical studies that indicate it can be useful in immune regulation, wound repair, antioxidation, and as an anti-tumor agent (46–48). Due to its extensive biological properties, Glycyrrhiza polysaccharides have attracted increasing attention from the scientific community. Such research will serve as a solid foundation for potential future applications as an immune modulator in poultry. Therefore, we evaluated the effect of Glycyrrhiza polysaccharides on immune cell proliferation *in vitro*, and *in vivo* and antioxidant activity and immune enhancement of chickens given the Newcastle disease (ND) vaccine.

Lymphocyte proliferation is an important indicator of the cellular immune level of the body (49). The *in vitro* test of T and B cell function is short and the conditions are easy to control, so it is suitable for the initial screening of immune-enhancing drugs (50). In this test, the effects of GPS on chickens' peripheral blood lymphocytes and spleen lymphocytes was measured in vitro using MTT assays. The results show that the maximum safe concentration of GPS is 39.06 μg/mL. In the range of 2.44–39.06 μg/mL, GPS can significantly promote the proliferation of T and B lymphocytes. DCs are the most powerful known antigen presenting cells (APCs) (51). DCs can stimulate the activation and proliferation of naive T cells, while macrophages and B cells can only stimulate activated T cells or memory T cells (52). Therefore, DCs are the initiators of specific immune responses. Monocytes, macrophages, and DCs are all derived from myeloid common precursor cells (CMPs). Under the stimulation of GM-CSF and IL-4, CMPs can gradually differentiate into DCs with typical morphological characteristics and functions. The results of this experiment show that GPS in the range of 4.88–39.06 μg/mL can significantly promote the proliferation of chBM-DCs precursor cells, providing an immune enhancement effect. Th1 and Th2 are subsets of helper T cells and are therefore critical for immunomodulation. They can secrete a variety of cytokines and play a major role in the regulation of specific and non-specific immunity. Th1 cells are responsible for cellular immunity, secreting IL-2, TNF- α, IFN- γ, and IFN- α. Th2 cells generally participate in humoral immunity by secreting IL-4, IL-6, and IL-10 (53). IL-12 is a cytokine necessary for activating CD4+ T cells (54). IL-12 and IFN-γ can promote the differentiation of initial Th cells into the Th1 subset, and in turn, Th1 can secrete a large amount of IFN-γ, making it vital to the elimination of intracellular pathogens. TNF-α and IL-1 β are essential for the development of inflammation, and are responsible for activating leukocytes and upregulating endothelial cell adhesion molecules (55). IL-12p70 is the most important cytokine for inducing a Th1 response; it triggers NK and T cells to produce IFN-γ and enhances their cytolytic activity, effectively activating anti-tumor effector cells in the tumor environment (56). Our results show that GPS-1enhances immune responses by regulating the Th1-like response induced by lymphocytes and DCs, increasing the cytokines secreted by Th1 (IL-12, TNF- α, and IFN- γ), and promoting the secretion of IL-1 β and IL-12p70 by chBM-DCs.

Nitric oxide (NO) is a highly reactive free radical that acts as a secondary messenger, neurotransmitter, and an effector molecule (57). It has a wide range of physiological functions in the body, such as relaxing vascular smooth muscle, inhibiting platelet aggregation, regulating cerebral blood flow, mediating cytotoxic effects, and regulating the immune system (58). The results of this test show that GPS can significantly enhance the immune function of lymphocytes and DCs, and further promote the secretion of NO by DCs.

Many natural extracts are good sources of antioxidants. Polysaccharides have free radical scavenging abilities that indue them with antioxidant and anti-aging properties (59). With an increased concentration of GPS-1 polysaccharide extract, the clearance rates of OH-, DPPH, ABTS and O^2−^ increased, respectively, reducing the damage to RAW264.7 cells caused by H_2_O_2_.

Different adjuvants in the ND vaccine can induce different immune responses in Lohmann Brown chickens. Humoral and cellular immune responses play an important role in host defense against ND infection. Antibodies are protective proteins produced by the body in response to an antigen, and antibody titers reflect the state of humoral immunity (60). Antibodies against ND usually appear within 6–10 days after infection and high levels of antibodies are correlated with host protection. Merz et al. reported that humoral and cellular immunity play an important role in host defense against NDV infection (60). This cellular immune response plays a leading role in immune protection against many avian infectious diseases (61). IL-2 and IFN-γ are both secreted by Th1, and possess complementary capabilities. IL-2 plays a key role in T cell differentiation and proliferation (62), while IFN-γ is critical to innate and acquired immunity against viral and intracellular bacterial and protozoan infections (63). Immune organs provide a place for immune cells to differentiate, develop and mature. The increase and decrease of the size of immune organs generally reflects the immune status of organisms. Recent studies have shown that the effects of polysaccharides on the size of the immune organs of chickens are mainly found in the spleen, thymus, and bursa of Fabricius (64). Our results show that GPS-1 can effectively amplify the immune response to Newcastle disease vaccination, cytokine secretion ability, and the development of immune organs in Lohmann Brown chickens.

## Conclusion

Based on our findings, we can conclude that GPS-1 can significantly promote the proliferation of immune cells and enhance the secretion of IL-12, IFN-γ, and TNF-α by lymphocytes and the secretion of IL-1 β, IL-12, IL-12P70, IFN-γ, TNF-α, and NO by dendritic cells. Both doses (1, 2, 4mg/ml, 1ml each chickens)of GPS-1 could significantly increase the body weight and serum antibody titer of Lohmann Brown chickens, enhance the secretion of IL-2 and IFN- γ in serum, and promote the development of immune organs. Our experiment also revealed that GPS-1 has a notable ability to scavenge free radicals, among them DPPH, hydrogen peroxide, ABTS, and superoxide anion radicals. Such scavenging abilities may prove useful in providing an immuno-supportive, antioxidant effect. GPS-1 has a clear protective effect against oxidative damage to RAW 264.7 cells. The results of this study indicated that GPS-1 can enhance immune regulation *in vitro* and *in vivo*, and antioxidation *in vitro*. A further study could assess the long-term effects and structure-activity relationships of GPS-1. More investigation into GPS-1 would also help establish a greater degree of accuracy regarding its therapeutic potential.

## Data availability statement

The datasets presented in this article are not readily available because no restrictions. Requests to access the datasets should be directed to YiW, wuyi2001cn@163.com.

## Ethics statement

The animal study was reviewed and approved by Nanjing Agricultural University No. PZ2020101. Written informed consent was obtained from the owners for the participation of their animals in this study.

## Author contributions

HZ: writing—original draft preparation and visualization. XC and NL: data curation and visualization. CDa: conceptualization and supervision. TZ: writing—original draft preparation. YC: writing—reviewing and methodology. KD: reviewing and editing. LY: data curation. AN and CDe: writing—reviewing and editing. XW and YuW: data validation. KL and YL: writing—reviewing. YiW: conceptualization, funding acquisition, project administration, and supervision. All authors contributed to the article and approved the submitted version.

## Funding

This research was financially supported by the National Natural Science Foundation of China (NSFC, Grant Nos. 31872514 and 32172900), the Open Project Program of Beijing Key Laboratory of Traditional Chinese Veterinary Medicine at Beijing University of Agriculture (No. kf-tcvm202101), Yunnan Provincial Science and Technology Department-Applied Basic Research Joint Special Funds of Yunnan University of Chinese Medicine [2018FF001(-020) and 2019FF002(-012)] and a project funded by the Priority Academic Program Development of Jiangsu Higher Education Institutions (PAPD).

## Conflict of interest

Author KD was employed by China Tobacco Henan Industrial Co., Ltd. The remaining authors declare that the research was conducted in the absence of any commercial or financial relationships that could be construed as a potential conflict of interest.

## Publisher's note

All claims expressed in this article are solely those of the authors and do not necessarily represent those of their affiliated organizations, or those of the publisher, the editors and the reviewers. Any product that may be evaluated in this article, or claim that may be made by its manufacturer, is not guaranteed or endorsed by the publisher.

## Nomenclature

GPS-1, Glycyrrhiza polysaccharide extract 1; ND, Newcastle disease; NDV, Newcastle disease vaccine; PHA-P, Phytin; mGM-CSF, Chicken recombinant proteins GM-CSF; il-4, Interleukin 4; chBM-DCs, immature chicken bone marrow dendritic cells.
